# Globally fixed-time synchronization of coupled neutral-type neural network with mixed time-varying delays

**DOI:** 10.1371/journal.pone.0191473

**Published:** 2018-01-25

**Authors:** Mingwen Zheng, Lixiang Li, Haipeng Peng, Jinghua Xiao, Yixian Yang, Yanping Zhang, Hui Zhao

**Affiliations:** 1 School of Science, Beijing University of Posts and Telecommunications, Beijing 100876, China; 2 School of Mathematics and Statistics, Shandong University of Technology, Zibo 255000, China; 3 Information Security Center, State Key Laboratory of Networking and Switching Technology, National Engineering Laboratory for Disaster Backup and Recovery, Beijing University of Posts and Telecommunications, Beijing 100876, China; 4 State Key Laboratory of Information Photonics and Optical Communications, Beijing University of Posts and Telecommunications, Beijing 100876, China; 5 Shandong Provincial Key Laboratory of Network Based Intelligent Computing, School of Information Science and Engineering, University of Jinan, Jinan 250022, China; East China Normal University, CHINA

## Abstract

This paper mainly studies the globally fixed-time synchronization of a class of coupled neutral-type neural networks with mixed time-varying delays via discontinuous feedback controllers. Compared with the traditional neutral-type neural network model, the model in this paper is more general. A class of general discontinuous feedback controllers are designed. With the help of the definition of fixed-time synchronization, the upper right-hand derivative and a defined simple Lyapunov function, some easily verifiable and extensible synchronization criteria are derived to guarantee the fixed-time synchronization between the drive and response systems. Finally, two numerical simulations are given to verify the correctness of the results.

## Introduction

The artificial neural network(ANN) is a very active frontier interdisciplinary, which has strong engineering application background and great research potential. It has been widely used in intelligent computing, pattern recognition, signal processing, associative memory, automatic control, and so on [1–5]. In the process of using large-scale integrated circuits to form a neural network, it inevitably leads to the emergence of various time-delays. These time-delays occur not only in the current states of the system, but also in the derivatives of the past states, i.e. there exists a neutral behavior in some systems. Scientific experiments show that the neutral behaviors have a great impact on the system, which has prompted lots of scholars to study the dynamics of neutral-type neural networks [6–11]. Furthermore, the synchronization of the coupled system, such as coupled oscillators, is a basic phenomenon of nonlinear dynamics. A series of papers have already studied the dynamic behaviors of coupled nonlinear oscillators, such as the dynamic behaviors of coupled Kuramoto oscillators with time delay [12], chimera states of [13, 14], coexistence phenomena of coherence and incoherence [15], etc [16].

Along with mathematical models of various kinds of neural networks have been put forward, the dynamic behavior of their structures and characteristics, such as the existence of the equilibrium points, the stability and boundedness of solutions, have attracted the wide attention and research of many scholars in many fields [17–21]. In the real world, the synchronous discharge of neurons is a universal phenomenon. For example, the synchronization in the visual cortex of conscious monkeys [22], the synchronization of the hippocampus and the cerebral cortex during the maze task [23], the synchronization of local brain regions in patients with Parkinson’s disease [24], the synchronization of neurons in circadian clock [25–28], and so forth [29, 30]. Neural networks have more complex dynamic characteristics and chaos phenomena. This naturally has made a lot of scholars to transform from the study of chaotic synchronization to neural network synchronization. Synchronization problem can be seen as an extension of the stability problem. And synchronization is a behavior of two or more dynamical systems under the action of external driving or mutual coupling, and constantly adjusts their dynamic characteristics to form a certain kind of overall consistency. At present, the main control methods to achieve synchronization have drive-response control [31–36], feedback control [37], adaptive control [38], impulsive control [39], intermittent control [40], sliding control [41] and pinning control [42]. And the synchronization forms mainly include complete synchronization [43, 44], lag synchronization [45–47], generalized synchronization [48, 49], etc [50, 51].

In many practical problems, finite-time synchronization is of interests rather than the synchronization over infinite time. Here, there are two ways of understanding. One is finite-time synchronization that means the system converges within a finite-time interval for given any initial value, and different initial values result in different synchronization time; the other is fixed-time synchronization that means the convergence time has a uniform upper-bounds for all initial values within a defined interval. About the finite-time synchronization of neural networks, there has existed some literatures on this study. Ref. [52] investigated the finite-time synchronization problem of a class of neutral complex dynamical networks with Markovian switching by using pinning control technique. Ref. [53] studied the finite-time synchronization for a class of the complex dynamical network with non-derivative and derivative coupling and proposed a new finite-time synchronization theory. Refs. [54, 55] discussed the finite-time synchronization problem of a class of complex dynamical network with coupled items. Because the initial values of many practical systems are difficult to determine, the final settling time in finite-time synchronization is not easy to be obtained. The fixed-time synchronization theory can overcome this shortcoming. However, the references about the fixed-time synchronization are relatively less. Ref. [56] studied the fixed-time master-slave synchronization of Cohen-Grossberg neural networks, which contains only one kind of time-varying delay *τ*(*t*) and has not coupling items. Ref. [57] studied the fixed-time synchronization control protocol of multi-agent systems. Refs. [58–62] mainly focused on the fixed-time stability of some simple nonlinear systems. For example, Ref. [61] considered the finite-time and fixed-time stability control problems of linear multi-input system. But, there are few studies on the fixed-time synchronization of coupling neutral-type neural networks with mixed time-varying delays.

Through the above discussions, the motivation of our research is summarized as follows: (1) in the theory aspect, there is little research on the fixed-time synchronization problem of dynamical neural network, especially the fixed-time synchronization of coupled neutral neural networks with mixed delays; (2) in the application aspect, the fixed-time synchronization is more suitable for practical application than the finite-time synchronization or asymptotic synchronization. Because the settling time of the finite-time synchronization depends on the initial value of the system, but the initial value is not easy to obtain. And the settling time of asymptotic synchronization may be infinite. Motivated by the above discussions, this paper studies the globally fixed-time synchronization control problem for the drive-response coupling neutral-type neural network with mixed time-varying delays, which can achieve synchronization in fixed time independent of the initial values. It has not been studied in the existing references. The proposed coupled neutral-type neural networks is general and many models can be considered as a special case of this model [7, 63–65]. According to the proposed model, we design a general nonlinear and discontinuous control law which involves a variety of different time-delays. During the proof process of the conclusion, a simple Lyapunov function is constructed. With the aid of upper right-hand derivative, the definition of fixed-time synchronization, and related lemmas, we obtain simple synchronization criteria of the drive-response coupled neutral-type neural networks with mixed time-varying delays. The main contributions are outlined as follows:

(1) The globally fixed-time synchronization problem for the drive-response coupling neutral-type neural networks with mixed time-varying delays is studied. At present, there are few studies on the globally fixed-time synchronization problem of coupled neutral-type neural networks.

(2) The network model we propose has not only neutral-type time-varying delays and discrete time-varying delays, but also distributed time-varying delays. In addition, the coupling term is also included in our model. Therefore, the obtained results in this paper are more general than the aforementioned works.

(3) We design a class of new and general discontinuous fixed-time synchronization feedback controllers, define a simple Lyapunov function and derive some easily verifiable and extensible synchronization criteria to achieve the fixed-time synchronization of drive-response systems. The sufficient conditions in our results are more simple and easier to calculate than other methods(such as LMI method).

(4) Two simulation examples are given to verify the validity of the main theorem and corollary.

The rest of this paper is organized as follows. Section 2 introduces the coupled neutral-type neural network model, some definitions and lemmas about fixed-time synchronization. Section 3 gives the fixed-time synchronization controller and derives the sufficient conditions to ensure the drive-response system to achieve fixed-time synchronization. Two numerical examples to verify the main results are given in Section 4. Finally, we summarize the paper and put forward the prospect in Section 5.

## Network model and preliminaries

In this section, we will give the mathematical model of the coupled neutral-type neural network, some assumptions, definitions, and lemmas about the fixed-time synchronization problem.

### Network model

Inspired by Ref. [66, 67], the mathematical model of coupled neutral-type neural network which contains *N* identical networks in this paper is given as follows:

$$\begin{array}{c} \begin{array}{cc} {\dot{x}}_{i}\left(t\right) & =d_{i}{\dot{x}}_{i}\left(t-\tau_{1}\left(t\right)\right)-c_{i}x_{i}\left(t\right)+\sum_{j=1}^{n}a_{ij}f\left(x_{i}\left(t\right)\right)\\ & +\sum_{j=1}^{n}b_{ij}g\left(x_{i}\left(t-\tau_{2}\left(t\right)\right)\right)+\sum_{j=1}^{n}h_{ij}\int_{t-\tau_{3}\left(t\right)}^{t}q\left(x_{i}\left(s\right)\right)ds\\ & +\kappa \sum_{l=1}^{N}m_{il}\Gamma x_{l}\left(t\right)+I_{i},i=1,2,\cdots ,N, \end{array} \end{array}$$ (1)

with initial conditions

$$\begin{array}{c} x_{i}\left(t\right)=\phi \left(t\right),t\in \left[-\tau ,0\right],\\ \tau =\max_{t\in [-\tau ,0]}\left\{\tau_{1}\left(t\right),\tau_{2}\left(t\right),\tau_{3}\left(t\right)\right\}, \end{array}$$

where *x*_*i*_(*t*) = [*x*_*i*1_(*t*), *x*_*i*2_(*t*), ⋯, *x*_*in*_(*t*)]^*T*^ is the state variable of the *i*th neutral-type neural network. *D* = diag{*d*_1_, *d*_2_, ⋯, *d*_*n*_}, *A* = (*a*_*ij*_)_*n*×*n*_, *B* = (*b*_*ij*_)_*n*×*n*_, *H* = (*h*_*ij*_)_*n*×*n*_ are state time-varying delays feedback connection weight matrices of the neurons, respectively, and *τ*_1_(*t*), *τ*_2_(*t*), *τ*_3_(*t*) are time-varying delays. *C* = diag{*c*_1_, *c*_2_, ⋯, *c*_*n*_} is the state self-feedback diagonal matrix. *I* = [*I*_1_, *I*_2_, ⋯, *I*_*N*_]^*T*^ is the external input vector. *f*(*x*) = [*f*(*x*_*i*1_(*t*)), *f*(*x*_*i*2_(*t*)), ⋯, *f*(*x*_*in*_(*t*))]^*T*^, *g*(*x*) = [*g*(*x*_*i*1_(*t*)), *g*(*x*_*i*2_(*t*)), ⋯, *g*(*x*_*in*_(*t*))]^*T*^, *q*(*x*) = [*q*(*x*_*i*1_(*t*)), *q*(*x*_*i*2_(*t*)), ⋯, *q*(*x*_*in*_(*t*))]^*T*^ are activation functions. *κ* denotes the coupling strength. *M* = (*m*_*il*_)_*N*×*N*_ is the outer coupling matrix defined as follows: if there exist communications between two neural networks via an edge, then *m*_*il*_ = 1; otherwise, *m*_*il*_ = 0, *i* ≠ *l*, meanwhile, *m*_*il*_ = *m*_*li*_. And *M* satisfies dissipation condition, i.e. $m_{ii}=-\sum_{l=1,l\neq i}^{N}m_{il}$. Γ = diag{*γ*_1_, *γ*_2_, ⋯, *γ*_*n*_} is the inner coupling matrix, where *γ*_*i*_ > 0, *i* = 1, 2, ⋯, *n*.

Let the network model (1) be the drive system, and the corresponding response system is formulated as

$$\begin{array}{c} \begin{array}{cc} {\dot{y}}_{i}\left(t\right) & =d_{i}{\dot{y}}_{i}\left(t-\tau_{1}\left(t\right)\right)-c_{i}y_{i}\left(t\right)+\sum_{j=1}^{n}a_{ij}f\left(y_{i}\left(t\right)\right)\\ & +\sum_{j=1}^{n}b_{ij}g\left(y_{i}\left(t-\tau_{2}\left(t\right)\right)\right)+\sum_{j=1}^{n}h_{ij}\int_{t-\tau_{3}\left(t\right)}^{t}q\left(y_{i}\left(s\right)\right)ds\\ & +\kappa \sum_{l=1}^{N}m_{il}\Gamma y_{l}\left(t\right)+I_{i}+u_{i}\left(t\right), \end{array} \end{array}$$ (2)

with initial conditions

$$\begin{array}{c} y_{i}\left(t\right)=\varphi \left(y_{i}\left(t\right)\right),t\in \left[-\tau ,0\right],\\ \tau =\max_{t\in [-\tau ,0]}\left\{\tau_{1}\left(t\right),\tau_{2}\left(t\right),\tau_{3}\left(t\right)\right\}, \end{array}$$

where *y*_*i*_(*t*) = [*y*_*i*1_(*t*), *y*_*i*2_(*t*), ⋯, *y*_*in*_(*t*)]^*T*^ is the state variable of the *i*th neutral-type neural network. *u*_*i*_(*t*) = [*u*_*i*1_, *u*_*i*2_, ⋯, *u*_*in*_]^*T*^ is the controller to be designed in main results. The other parameters are the same as the model (1).

For the parameters of drive-response systems (1) and (2) throughout this paper, we introduce the following assumptions.

*Assumption 1*. The activation functions *f*(*x*), *g*(*x*), *q*(*x*) are Lipschitz continuous, i.e. there exist Lipschitz constants *f*_*i*_, *g*_*i*_, *q*_*i*_, *i* = 1, 2, ⋯, *n* satisfying the following conditions.

$$\begin{array}{c} \begin{array}{cc} & |f\left(y_{i}\right)-f\left(x_{i}\right)|\leq f_{i}\left|y_{i}-x_{i}\right|,\\ & |g\left(y_{i}\right)-g\left(x_{i}\right)|\leq g_{i}\left|y_{i}-x_{i}\right|,\\ & |q\left(y_{i}\right)-q\left(x_{i}\right)|\leq q_{i}\left|y_{i}-x_{i}\right|. \end{array} \end{array}$$

And *F* = diag{*f*_1_, *f*_2_, ⋯, *f*_*n*_}, *G* = diag{*g*_1_, *g*_2_, ⋯, *g*_*n*_}, *Q* = diag{*q*_1_, *q*_2_, ⋯, *q*_*n*_}.

Denote the error system *e*_*i*_(*t*) = *y*_*i*_(*t*) − *x*_*i*_(*t*), and the dynamical equation of *e*_*i*_(*t*) can be expressed as

$$\begin{array}{c} \begin{array}{cc} {\dot{e}}_{i}\left(t\right) & =d_{i}{\dot{e}}_{i}\left(t-\tau_{1}\left(t\right)\right)-c_{i}e_{i}\left(t\right)+\sum_{j=1}^{n}a_{ij}\left(f\left(y_{i}\left(t\right)\right)-f\left(x_{i}\left(t\right)\right)\right)\\ & +\sum_{j=1}^{n}b_{ij}(g\left(y_{i}\left(t-\tau_{2}\left(t\right)\right)-g\left(x_{i}\left(t-\tau_{2}\left(t\right)\right)\right)\right)\\ & +\sum_{j=1}^{n}h_{ij}\int_{t-\tau_{3}\left(t\right)}^{t}\left(q\left(y_{i}\left(s\right)\right)-q\left(x_{i}\left(s\right)\right)\right)ds\\ & +\kappa \sum_{l=1}^{N}m_{il}\Gamma e_{l}\left(t\right)+u_{i}\left(t\right), \end{array} \end{array}$$ (3)

with the initial conditions

$$\begin{array}{c} \begin{array}{cc} & e_{i}\left(t\right)=\varphi \left(t\right)-\phi \left(t\right),t\in \left[-\tau ,0\right],\\ & \tau =\max_{t\in [-\tau ,0]}\left\{\tau_{1}\left(t\right),\tau_{2}\left(t\right),\tau_{3}\left(t\right)\right\}. \end{array} \end{array}$$

### Definitions and lemmas

In this subsection, we will introduce some definitions and lemmas related to the fixed-time synchronization. They are necessary in the process of derivation of the main results.

Suppose the origin be an equilibrium of (3) (if the equilibrium is not origin, we can move the equilibrium point to the origin through the translation transformation.)

*Definition 1*. ([60]) The origin of system (3) is said to be globally uniformly finite-time stable if it is globally uniformly asymptotically stable and there exists a locally bounded function $T:C^{n}\left[-\tau ,0\right]\to R_{+}\cup \left\{0\right\}$, such that *e*(*t*, *e*_0_(*t*)) = 0 for all *t* ≥ *T*(*e*_0_(*t*)), where *e*(⋅, *e*_0_(*t*)) is an arbitrary solution of the error system (3). The function *T* is called the settling-time function.

*Definition 2*. ([59]) The origin of error system (3) is said to be globally fixed-time stable if it is globally uniformly finite-time stable and the settling-time *T* is globally bounded, i.e. $\exists T_{max}\in R_{+}$ such that $T\left(e_{0}\left(t\right)\right)\leq T_{max},\forall e_{0}\left(t\right)\in R^{n}.$

*Definition 3*. If *e*(*t*) is Lyapunov stable, then the drive-response systems (1) and (2) are said to achieve globally fixed-time synchronization if there exists *T*(*e*_0_(*t*)) in some finite time such that

$$\{\begin{array}{l} \underset{t\to T\left(e_{0}\left(t\right)\right)}{\lim}\|e\left(t\right)\|=0,\\ e\left(t\right)=0,\forall t\geq T\left(e_{0}\left(t\right)\right)\\ T\left(e_{0}\left(t\right)\right)\leq T_{max},\forall e_{0}\left(t\right)\in {\mathbb{C}}^{n}\left[-\tau ,0\right]. \end{array}$$

*Remark 1*. In the *Definition 1*, *e*(*t*) = 0 ⇒ *y*(*t*) = *x*(*t*), *u*_*i*_(*t*) to be designed is a function of *e*_*i*_(*t*) and *u*_*i*_(*t*) = 0 when *e*_*i*_(*t*) = 0.

*Remark 2*. According to the *Definition 1* and *Definition 2*, we can see the main difference between finite-time stability and fixed-time stability is whether the settling time is independent to the initial value. The settling-time of fixed-time stability is independent to the initial value.

*Remark 3*. From the *Definition 2* and *Definition 3*, we can conclude the globally fixed-time stability of system (3) is equivalent to the fixed-time synchronization of systems (1) and (2).

**Lemma 1**. [59] If there exists a continuous radically unbounded function $V:R^{n}\to R_{+}\cup \left\{0\right\}$ such that (1) *V*(*x*) = 0 ⇒ *x* = 0. (2) Any solution *e*(*t*) of system (3) satisfies

$$\begin{array}{c} \dot{V}\left(e\left(t\right)\right)\leq -aV^{p}\left(e\left(t\right)\right)-bV^{q}\left(e\left(t\right)\right), \end{array}$$ (4)

for some *a*, *b* > 0, *p* > 1, 0 < *q* < 1, where $\dot{V}$ denotes the derivative of *V*. Then,

$$\begin{array}{c} V\left(e\left(t\right)\right)=0,t\geq T\left(e_{0}\right), \end{array}$$

with the settling time bounded by

$$\begin{array}{c} T\left(e_{0}\right)\leq T_{max}=\frac{1}{a(p-1)}+\frac{1}{b(1-q)}. \end{array}$$ (5)

**Lemma 2**. [68] If there exists a continuous radically unbounded function $V:R^{n}\to R_{+}\cup \left\{0\right\}$ such that 1) *V*(*x*) = 0 ⇒ *x* = 0. 2) Any solution *e*(*t*) of system (3) satisfies

$$\begin{array}{c} \dot{V}\left(e\left(t\right)\right)\leq -aV^{p}\left(e\left(t\right)\right)-bV^{q}\left(e\left(t\right)\right) \end{array}$$ (6)

for some $a,b>0,p=1-\frac{1}{2\rho },q=1+\frac{1}{2\rho },\rho >1$, where $\dot{V}$ denotes the derivative of *V*. Then the origin is globally fixed-time stable for system (3) and more exact estimation of the settling time can be obtained as

$$\begin{array}{c} T\left(e_{0}\right)\leq T_{max}=\frac{\pi \rho }{\sqrt{ab}}. \end{array}$$ (7)

**Lemma 3**. [69] Let *a*_1_, *a*_2_, ⋯, *a*_*N*_ ≥ 0, 0 < *p* ≤ 1, *q* > 1, then the following two inequalities hold

$$\begin{array}{c} \sum_{i=1}^{N}a_{i}^{p}\geq {\left(\sum_{i=1}^{N}a_{i}\right)}^{p},\;\sum_{i=1}^{N}a_{i}^{q}\geq N^{1-q}{\left(\sum_{i=1}^{N}a_{i}\right)}^{q}. \end{array}$$

## Main results

In this section, we will design the controller *u*(*t*) and deduce some sufficient conditions in order to achieve fixed-time synchronization of neutral-type neural networks (1) and (2).

The nonlinear controller in response system (2) is designed as follows:

$$\begin{array}{c} \begin{array}{cc} u_{i}\left(t\right) & =-sign\left(e_{i}\left(t\right)\right)(\xi_{i}|e_{i}\left(t\right)|+\omega_{1i}\left|{\dot{e}}_{i}\left(t-\tau_{1}\left(t\right)\right)\right|\\ & +\omega_{2i}|e_{i}\left(t-\tau_{2}\left(t\right)\right)|+\omega_{3i}\int_{t-|\tau_{3}\left(t\right)|}^{t}\left|e_{i}\left(s\right)\right|ds\\ & +k_{i}|e_{i}{\left(t\right)|}^{\alpha }+r_{i}|e_{i}\left(t\right){|}^{\beta }), \end{array} \end{array}$$ (8)

where *sign*(⋅) denotes sign function, *Ξ* = diag{*ξ*_1_, *ξ*_2_, ⋯, *ξ*_*n*_}, Ω_1_ = diag{*ω*_11_, *ω*_12_, ⋯, *ω*_1*N*_}, Ω_2_ = diag{*ω*_21_, *ω*_22_, ⋯, *ω*_2*N*_}, Ω_3_ = diag{*ω*_31_, *ω*_32_, ⋯, *ω*_3*N*_}, *K* = diag{*k*_1_, *k*_2_, ⋯, *k*_*n*_}, *R* = diag{*r*_1_, *r*_2_, ⋯, *r*_*n*_}, *α* > 1, 0 < *β* < 1 are constants.

Replacing error system (3) with *u*_*i*_(*t*) and according to *Assumption 1*, we have

$$\begin{array}{c} \begin{array}{cc} {\dot{e}}_{i}\left(t\right) & =d_{i}{\dot{e}}_{i}\left(t-\tau_{1}\left(t\right)\right)-c_{i}e_{i}\left(t\right)+\sum_{j=1}^{n}a_{ij}\left(f\left(y_{i}\left(t\right)\right)-f\left(x_{i}\left(t\right)\right)\right)\\ & +\sum_{j=1}^{n}b_{ij}(g\left(y_{i}\left(t-\tau_{2}\left(t\right)\right)-g\left(x_{i}\left(t-\tau_{2}\left(t\right)\right)\right)\right)\\ & +\sum_{j=1}^{n}h_{ij}\int_{t-\tau_{3}\left(t\right)}^{t}\left(q\left(y_{i}\left(s\right)\right)-q\left(x_{i}\left(s\right)\right)\right)ds\\ & +\kappa \sum_{l=1}^{N}m_{il}\Gamma e_{l}\left(t\right)+u_{i}\left(t\right)\\ & \leq d_{i}{\dot{e}}_{i}\left(t-\tau_{1}\left(t\right)\right)-c_{i}e_{i}\left(t\right)+\sum_{j=1}^{n}a_{ij}f_{i}e_{i}\left(t\right)\\ & +\sum_{j=1}^{n}b_{ij}g_{i}e_{i}\left(t-\tau_{2}\left(t\right)\right)+\sum_{j=1}^{n}h_{ij}q_{i}\int_{t-\tau_{3}\left(t\right)}^{t}e_{i}\left(s\right)ds\\ & +\kappa \sum_{l=1}^{N}m_{il}\Gamma e_{l}\left(t\right)-sign\left(e_{i}\left(t\right)\right)(\xi_{i}\left|e_{i}\left(t\right)\right|\\ & +\omega_{1i}|{\dot{e}}_{i}\left(t-\tau_{1}\left(t\right)\right)|+\omega_{2i}\left|e_{i}\left(t-\tau_{2}\left(t\right)\right)\right|\\ & +\omega_{3i}\int_{t-|\tau_{3}\left(t\right)|}^{t}|e_{i}\left(s\right)|ds+k_{i}|e_{i}{\left(t\right)|}^{\alpha }+r_{i}{\left|e_{i}\left(t\right)\right|}^{\beta }). \end{array} \end{array}$$ (9)

**Theorem 1**. Suppose *Assumption 1* holds, then the drive-response systems (1) and (2) can achieve globally fixed-time synchronization under the controller (8) if the following conditions hold:

$$\{\begin{array}{l} \sum_{j=1}^{n}a_{ij}f_{i}-c_{i}-\xi_{i}<0,\\ d_{i}-\omega_{1i}<0,\\ \sum_{j=1}^{n}b_{ij}g_{i}-\omega_{2i}<0,\\ \sum_{j=1}^{n}h_{ij}q_{i}-\omega_{3i}<0,\\ i=1,2,\cdots ,N. \end{array}$$ (10)

Moreover,

$$\begin{array}{c} \lim_{t\to T_{max}}\left\|e\left(t\right)\right\|=0,e\left(t\right)=0,\forall t\geq T_{max}, \end{array}$$

and the settling time given as

$$\begin{array}{c} \begin{array}{cc} T_{max} & =\frac{1}{(\alpha -1)\;{\text{min}}_{1\leq i\leq N}\left\{k_{i}N^{\frac{1-\alpha }{2}}\right\}}\\ & +\frac{1}{(1-\beta )\;{\text{min}}_{1\leq i\leq N}\left\{r_{i}\right\}}. \end{array} \end{array}$$ (11)

**Proof**. Consider a Lyapunov function defined by

$$\begin{array}{c} V\left(e_{i}\left(t\right)\right))=\sum_{i=1}^{N}\left|e_{i}\left(t\right)\right|. \end{array}$$ (12)

Calculate the upper right-hand derivative of *V*(*e*_*i*_(*t*)) along the error system (3) and replace the inequality (9) with ${\dot{e}}_{i}\left(t\right)$, we can obtain

$$\begin{array}{ccc} {\dot{V}}^{+}\left(e_{i}\left(t\right)\right) & & =\sum_{i=1}^{N}sign\left(e_{i}\left(t\right)\right){\dot{e}}_{i}\left(t\right)\\ & & \leq \sum_{i=1}^{N}sign\left(e_{i}\left(t\right)\right)(d_{i}{\dot{e}}_{i}\left(t-\tau_{1}\left(t\right)\right)-c_{i}e_{i}\left(t\right)+\sum_{j=1}^{n}a_{ij}f_{i}e_{i}\left(t\right)\\ & & +\sum_{j=1}^{n}b_{ij}g_{i}e_{i}\left(t-\tau_{2}\left(t\right)\right)+\sum_{j=1}^{n}h_{ij}q_{i}\int_{t-\tau_{3}\left(t\right)}^{t}e_{i}\left(s\right)ds+\kappa \sum_{l=1}^{N}m_{il}\Gamma e_{l}\left(t\right)\\ & & -sign\left(e_{i}\left(t\right)\right)(\xi_{i}|e_{i}\left(t\right)|+\omega_{1i}|{\dot{e}}_{i}\left(t-\tau_{1}\left(t\right)\right)|+\omega_{2i}\left|e_{i}\left(t-\tau_{2}\left(t\right)\right)\right|\\ & & +\omega_{3i}\int_{t-|\tau_{3}\left(t\right)|}^{t}|e_{i}\left(s\right)|ds+k_{i}|e_{i}{\left(t\right)|}^{\alpha }+r_{i}{\left|e_{i}\left(t\right)\right|}^{\beta }))\\ & & \leq \sum_{i=1}^{N}d_{i}|{\dot{e}}_{i}\left(t-\tau_{1}\left(t\right)\right)|-\sum_{i=1}^{N}c_{i}\left|e_{i}\left(t\right)\right|\\ & & +\sum_{i=1}^{N}\sum_{j=1}^{n}a_{ij}f_{i}|e_{i}\left(t\right)|+\sum_{i=1}^{N}\sum_{j=1}^{n}b_{ij}g_{i}\left|e_{i}\left(t-\tau_{2}\left(t\right)\right)\right|\\ & & +\sum_{i=1}^{N}\sum_{j=1}^{n}h_{ij}q_{i}\int_{t-|\tau_{3}\left(t\right)|}^{t}|e_{i}\left(s\right)|ds+\kappa \sum_{i=1}^{N}\sum_{l=1}^{N}m_{il}\Gamma |e_{l}\left(t\right)|-\sum_{i=1}^{N}\xi_{i}\left|e_{i}\left(t\right)\right|\\ & & -\sum_{i=1}^{N}\omega_{1i}|{\dot{e}}_{i}\left(t-\tau_{1}\left(t\right)\right)|-\sum_{i=1}^{N}\omega_{2i}\left|e_{i}\left(t-\tau_{2}\left(t\right)\right)\right|\\ & & -\sum_{i=1}^{N}\omega_{3i}\int_{t-|t_{3}\left(t\right)|}^{t}|e_{i}\left(s\right)|ds-\sum_{i=1}^{N}k_{i}|e_{i}{\left(t\right)|}^{\alpha }-\sum_{i=1}^{N}r_{i}{\left|e_{i}\left(t\right)\right|}^{\beta }\\ & & \leq \sum_{i=1}^{N}(\sum_{j=1}^{n}a_{ij}f_{i}-c_{i}-\xi_{i})\left|e_{i}\left(t\right)\right|\\ & & +\sum_{i=1}^{N}\left(d_{i}-\omega_{1i}\right)|{\dot{e}}_{i}\left(t-\tau_{1}\left(t\right)\right)|+\sum_{i=1}^{N}\left(\sum_{j=1}^{n}b_{ij}g_{i}-\omega_{2i}\right)\left|e\left(t-\tau_{2}\right)\right|\\ & & +\sum_{i=1}^{N}(\sum_{j=1}^{n}h_{ij}q_{i}-\omega_{3i})\int_{t-|\tau_{3}\left(t\right)|}^{t}\left|e_{i}\left(s\right)\right|ds\\ & & -\sum_{i=1}^{N}k_{i}|e_{i}{\left(t\right)|}^{\alpha }-\sum_{i=1}^{N}r_{i}{\left|e_{i}\left(t\right)\right|}^{\beta }. \end{array}$$

According to the conditions (10) and *Lemma 3*, we have

$$\begin{array}{ccc} {\dot{V}}^{+}\left(e\left(t\right)\right) & & \leq -\sum_{i=1}^{N}k_{i}|e_{i}^{\alpha }\left(t\right)|-\sum_{i=1}^{N}r_{i}\left|e_{i}^{\beta }\left(t\right)\right|\\ & & \leq -\underset{1\leq i\leq N}{\text{min}}\left\{k_{i}\right\}\sum_{i=1}^{N}|e_{i}\left(t\right){|}^{\alpha }-\underset{1\leq i\leq N}{\text{min}}\left\{r_{i}\right\}\sum_{i=1}^{N}{\left|e_{i}\right|}^{\beta }\\ & & \leq -\underset{1\leq i\leq N}{\text{min}}\left\{k_{i}N^{1-\alpha }\right\}V^{\alpha }\left(e\left(t\right)\right)-\underset{1\leq i\leq N}{\text{min}}\left\{r_{i}\right\}V^{\beta }\left(e\left(t\right)\right) \end{array}$$

Taking *a* = min_1≤*i*≤*N*_{*k*_*i*_*N*^1−*α*^}, *b* = min_1≤*i*≤*N*_{*r*_*i*_}, *p* = *α*, *q* = *β*, then *p* > 1, 0 < *q* < 1 and from *Lemma 3*, we have *V*(*e*(*t*)) = 0, *t* ≥ *T*_*max*_ and the settling time *T*_*max*_ is given as follows:

$$\begin{array}{c} \begin{array}{cc} T_{max} & =\frac{1}{(\alpha -1)\;{\text{min}}_{1\leq i\leq N}\left\{k_{i}N^{\frac{1-\alpha }{2}}\right\}}\\ & +\frac{1}{(1-\beta )\;{\text{min}}_{1\leq i\leq N}\left\{r_{i}\right\}}. \end{array} \end{array}$$

Thus, we complete the proof.

**Corollary 1**. According to **Lemma 2**., if $\alpha =1-\frac{1}{2\rho },\beta =1+\frac{1}{2\rho },\rho >1$, the setting time in **Theorem 1** can be rewritten as the following form:

$$\begin{array}{c} T_{max}=\frac{\pi \rho }{\sqrt{{\text{min}}_{1\leq i\leq N}\{k_{i}N^{1-\alpha }\}\;{\text{min}}_{1\leq i\leq N}\left\{r_{i}\right\}}}. \end{array}$$

*Remark 4*. Our proposed model is more general than the other models in some literatures [52, 56, 70]. When coupling strength *κ* = 0, model (1) is changed into the common neutral-type neural networks without coupling items. When *d*_*i*_ = 0, the model becomes the common neural networks without neutral items. For these special circumstances, we have the following corollaries.

**Corollary 2**. Suppose *Assumption 1* holds and the coupling strength *κ* = 0 in the drive-response system (1) and (2), then they can achieve a globally fixed-time synchronization under the controller (8) if the inequality conditions (10) hold.

*Remark 5*. In the proof of **Theorem 1**, the coupling item is removed according to the dissipativeness of coupling matrix *M*. Therefore, the information of coupling item is not included in the synchronization conditions of **Theorem 1** and **Corollary 2**.

When *d*_*i*_ = 0 in the drive-response systems (1) and (2), we define the following controller

$$\begin{array}{c} \begin{array}{cc} u_{i}\left(t\right) & =-sign\left(e_{i}\left(t\right)\right)(\xi_{i}|e_{i}\left(t\right)|+\omega_{2i}\left|e_{i}\left(t-\tau_{2}\left(t\right)\right)\right|\\ & +\omega_{3i}\int_{t-|\tau_{3}\left(t\right)|}^{t}|e_{i}\left(s\right)|ds+k_{i}|e_{i}{\left(t\right)|}^{\alpha }+r_{i}|e_{i}\left(t\right){|}^{\beta }). \end{array} \end{array}$$ (13)

**Corollary 3**. Suppose the *Assumption 1* holds and *d*_*i*_ = 0 in the drive-response system (1) and (2), then they can achieve globally fixed-time synchronization under the controller (13) if the following conditions hold:

$$\{\begin{array}{l} \sum_{j=1}^{n}a_{ij}f_{i}-c_{i}-\xi_{i}<0,\\ \sum_{j=1}^{n}b_{ij}g_{i}-\omega_{2i}<0,\\ \sum_{j=1}^{n}h_{ij}q_{i}-\omega_{3i}<0.\\ i=1,2,\cdots ,N. \end{array}$$ (14)

When *h*_*i*_ = 0 in the drive-response systems (1) and (2), we modify (8) as the following controller

$$\begin{array}{c} \begin{array}{cc} u_{i}\left(t\right) & =-sign\left(e_{i}\left(t\right)\right)(\xi_{i}|e_{i}\left(t\right)|+\omega_{1i}\left|{\dot{e}}_{i}\left(t-\tau_{1}\left(t\right)\right)\right|\\ & +\omega_{2i}|e_{i}\left(t-\tau_{2}\left(t\right)\right)|+k_{i}|e_{i}{\left(t\right)|}^{\alpha }+r_{i}|e_{i}\left(t\right){|}^{\beta }). \end{array} \end{array}$$ (15)

**Corollary 4**. Suppose *Assumption 1* holds and *h*_*i*_ = 0 in the drive-response system (1) and (2), then they can achieve the globally fixed-time synchronization under the controller (15) if the following conditions hold:

$$\{\begin{array}{l} \sum_{j=1}^{n}a_{ij}f_{i}-c_{i}-\xi_{i}<0,\\ d_{i}-\omega_{1i}<0,\\ \sum_{j=1}^{n}b_{ij}g_{i}-\omega_{2i}<0,\\ i=1,2,\cdots ,N. \end{array}$$ (16)

*Remark 6*. In the **Corollaries 1-3**, the settling time *T*_*max*_ is the same as that in **Theorem 1** because this two parameters *α*, *β* have not changed.

By modifying designed controller (8), we can obtain the fixed-time synchronization of the drive-response systems. The modified controller is given as follows

$$\begin{array}{c} \begin{array}{cc} u_{i}\left(t\right) & =-sign\left(e_{i}\left(t\right)\right)(\xi_{i}|e_{i}\left(t\right)|+\omega_{1i}\left|{\dot{e}}_{i}\left(t-\tau_{1}\left(t\right)\right)\right|\\ & +\omega_{2i}|e_{i}\left(t-\tau_{2}\left(t\right)\right)|+\omega_{3i}\int_{t-|\tau_{3}\left(t\right)|}^{t}|e_{i}\left(s\right)|ds+r_{i}|e_{i}\left(t\right){|}^{\beta }). \end{array} \end{array}$$ (17)

where 0 < *β* < 1, the other parameters are the same as those in the controller (8).

**Corollary 5**. Suppose *Assumption 1* holds and *h*_*i*_ = 0 in the drive-response systems (1) and (2), then they can achieve the globally finite-time synchronization under the controller (17) if the conditions (10) hold in the **Theorem 1**. Moreover,

$$\begin{array}{c} \lim_{t\to T_{max}}\left\|e\left(t\right)\right\|=0,e\left(t\right)=0,\forall t\geq T_{max}, \end{array}$$

and the settling time is given as

$$\begin{array}{c} T_{max}=\frac{\sum_{i=1}^{N}{\left|e_{i}\left(0\right)\right|}^{1-\beta }}{\left(1-\beta \right){\text{min}}_{1\leq i\leq N}\left\{r_{i}\right\}}. \end{array}$$

*Remark 7*. Compared with existing research on the fixed-time synchronization [56, 57, 71], although the theoretical framework of fixed-time is derived from the research of Polyakov et,al. [59–61], they study different system models. In this paper, we study the globally fixed-time synchronization of the coupling neutral-type neural networks with mixed time-varying delays, which no one seems to have studied such a model.

## Numerical examples

In order to verify the rightness of the theoretical results, we give two numerical examples.

*Example 1*: Consider the following two-dimensional neutral-type neural network with three identical neurons:

$$\begin{array}{c} \begin{array}{cc} {\dot{x}}_{i}\left(t\right) & =d_{i}{\dot{x}}_{i}\left(t-\tau_{1}\left(t\right)\right)-c_{i}x_{i}\left(t\right)+\sum_{j=1}^{2}a_{ij}f\left(x_{i}\left(t\right)\right)+\sum_{j=1}^{2}b_{ij}g\left(x_{i}\left(t-\tau_{2}\left(t\right)\right)\right)\\ & +\sum_{j=1}^{2}h_{ij}\int_{t-\tau_{3}\left(t\right)}^{t}q\left(x_{i}\left(s\right)\right)ds+\kappa \sum_{l=1}^{3}m_{il}\Gamma x_{l}\left(t\right)+I_{i},i=1,2,3. \end{array} \end{array}$$ (18)

The corresponding response system is shown below:

$$\begin{array}{c} \begin{array}{cc} {\dot{y}}_{i}\left(t\right) & =d_{i}{\dot{y}}_{i}\left(t-\tau_{1}\left(t\right)\right)-c_{i}y_{i}\left(t\right)+\sum_{j=1}^{2}a_{ij}f\left(y_{i}\left(t\right)\right)+\sum_{j=1}^{2}b_{ij}g\left(y_{i}\left(t-\tau_{2}\left(t\right)\right)\right)\\ & +\sum_{j=1}^{2}h_{ij}\int_{t-\tau_{3}\left(t\right)}^{t}q\left(y_{i}\left(s\right)\right)ds+\kappa \sum_{l=1}^{3}m_{il}\Gamma y_{l}\left(t\right)+I_{i}+u_{i}\left(t\right),i=1,2,3. \end{array} \end{array}$$ (19)

where the time-varying delays are *τ*_1_(*t*) = *τ*_2_(*t*) = 0.5|*sin*(*t*)|, *τ*_3_(*t*) = 0.5|*cos*(*t*)|, *f*(*e*_*i*_(*t*)) = *g*(*e*_*i*_(*t*)) = *h*(*e*_*i*_(*t*)) = *tanh*(*e*_*i*_(*t*)). Other parameters in the model (18) are chosen as follows:

$$\begin{array}{c} \begin{array}{cc} & D=\text{diag}\left\{0.6,0.8\right\},C=\text{diag}\left\{0.5,0.6\right\},A=[\begin{array}{cc} 0.2 & 0.3\\ 0.5 & 1 \end{array}],B=[\begin{array}{cc} 0.5 & 0.4\\ 0.6 & 0.3 \end{array}],\\ & H=[\begin{array}{cc} 0.3 & 0.2\\ 0.2 & 1 \end{array}],I={\left[0.1,0.1\right]}^{T},F=\text{diag}\left\{1,1\right\},G=\text{diag}\left\{1,1\right\},H=\text{diag}\left\{1,1\right\}\\ & M=[\begin{array}{ccc} -2 & 1 & 1\\ 1 & -2 & 1\\ 1 & 1 & -2 \end{array}],\Gamma =[\begin{array}{cc} 1 & 0\\ 0 & 1 \end{array}],\kappa =2. \end{array} \end{array}$$

According to the conditions (10) in **Theorem 1**, the parameters of controller (8) are set as follows:

$$\begin{array}{c} \begin{array}{cc} & \Xi =\text{diag}\left\{5,5\right\},\Omega_{1}=\text{diag}\left\{2,2\right\},\Omega_{2}=\text{diag}\left\{2,2\right\},\\ & \Omega_{3}=\text{diag}\left\{2,2\right\},K=\text{diag}\left\{4,3\right\},R=\text{diag}\left\{3,4\right\},\alpha =1.25,\beta =0.25. \end{array} \end{array}$$

The initial values of (18) and (19) are *x*_1_(*t*_0_) = [0.5 + *sin*(*t*), *cos*(*t*) − 0.5]^*T*^, *x*_2_(*t*_0_) = [*sin*(*t*) − 0.5, 0.5 + *cos*(*t*)]^*T*^, *x*_3_(*t*_0_) = [*sin*(*t*), *cos*(*t*)]^*T*^, *y*_1_(*t*_0_) = [0.1 + *sin*(*t*), *cos*(*t*) − 0.1]^*T*^, *y*_2_(*t*_0_) = [*sin*(*t*) − 0.1, 0.1 + *cos*(*t*)]^*T*^, *y*_3_(*t*_0_) = [0.5 + *sin*(*t*), *cos*(*t*) − 0.5]^*T*^.

**Fig 1:**
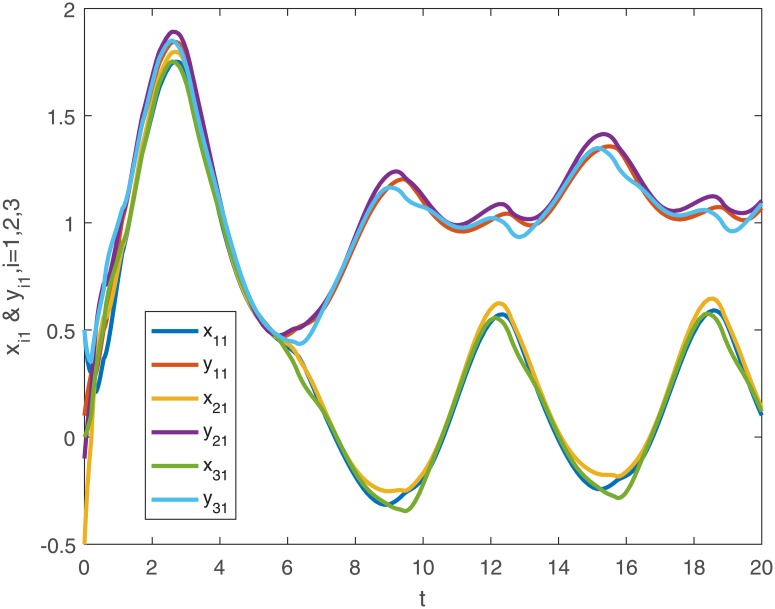
The first dimensional trajectories of the drive system (18) with initial conditions *x*_1_(*t*_0_) = [0.5 + *sin*(*t*), *cos*(*t*) − 0.5]^*T*^, *x*_2_(*t*_0_) = [*sin*(*t*) − 0.5, 0.5 + *cos*(*t*)]^*T*^, *x*_3_(*t*_0_) = [*sin*(*t*), *cos*(*t*)]^*T*^ and response system (19) with initial conditions *y*_1_(*t*_0_) = [0.1 + *sin*(*t*), *cos*(*t*) − 0.1]^*T*^, *y*_2_(*t*_0_) = [*sin*(*t*) − 0.1, 0.1 + *cos*(*t*)]^*T*^, *y*_3_(*t*_0_) = [0.5 + *sin*(*t*), *cos*(*t*) − 0.5]^*T*^ when no controller (8).

**Fig 2:**
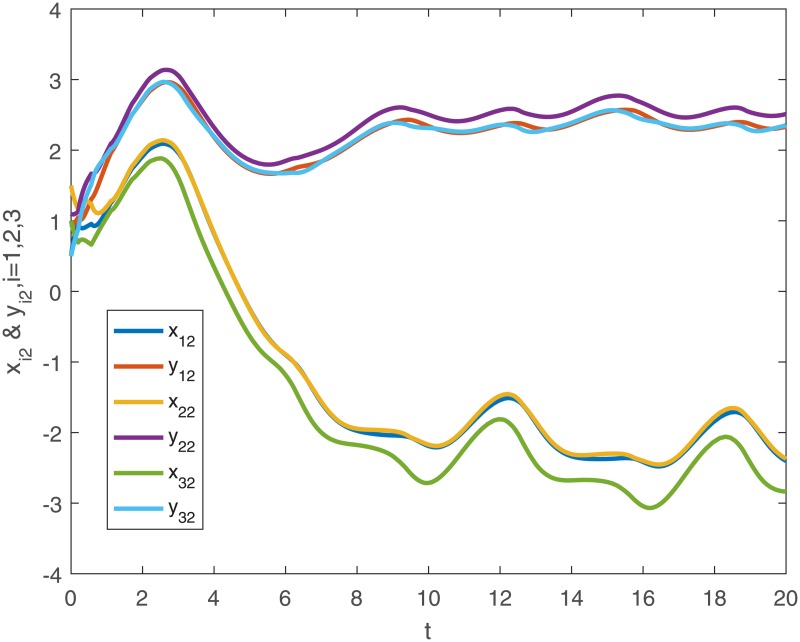
The second dimensional trajectories of the drive system (18) with initial conditions *x*_1_(*t*_0_) = [0.5 + *sin*(*t*), *cos*(*t*) − 0.5]^*T*^, *x*_2_(*t*_0_) = [*sin*(*t*) − 0.5, 0.5 + *cos*(*t*)]^*T*^, *x*_3_(*t*_0_) = [*sin*(*t*), *cos*(*t*)]^*T*^ and response system (19) with initial conditions *y*_1_(*t*_0_) = [0.1 + *sin*(*t*), *cos*(*t*) − 0.1]^*T*^, *y*_2_(*t*_0_) = [*sin*(*t*) − 0.1, 0.1 + *cos*(*t*)]^*T*^, *y*_3_(*t*_0_) = [0.5 + *sin*(*t*), *cos*(*t*) − 0.5]^*T*^ when no controller (8).

**Fig 3:**
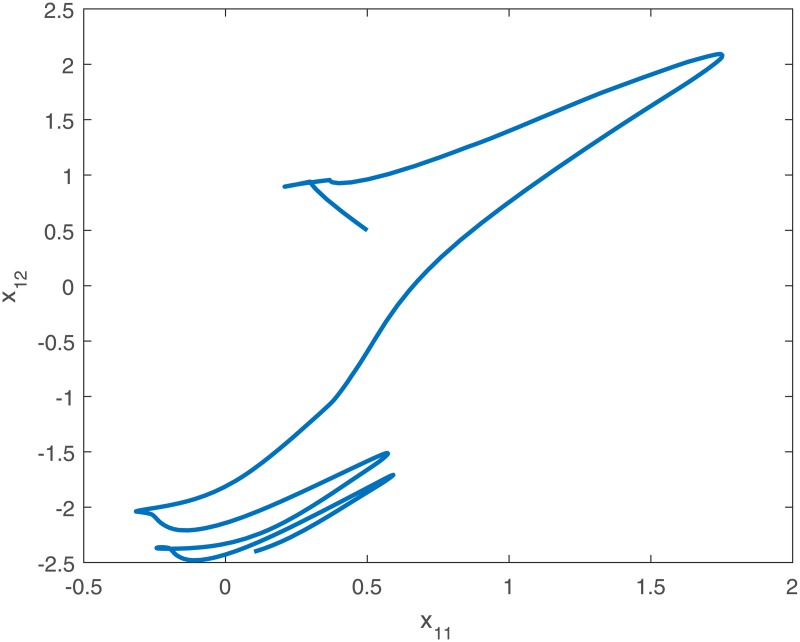
The phase diagram of the first neuron(*x*_11_ − *x*_12_) of the drive system (18) with initial conditions *x*_1_(*t*_0_) = [0.5 + *sin*(*t*), *cos*(*t*) − 0.5]^*T*^, *x*_2_(*t*_0_) = [*sin*(*t*) − 0.5, 0.5 + *cos*(*t*)]^*T*^, *x*_3_(*t*_0_) = [*sin*(*t*), *cos*(*t*)]^*T*^ when no controller (8).

**Fig 4:**
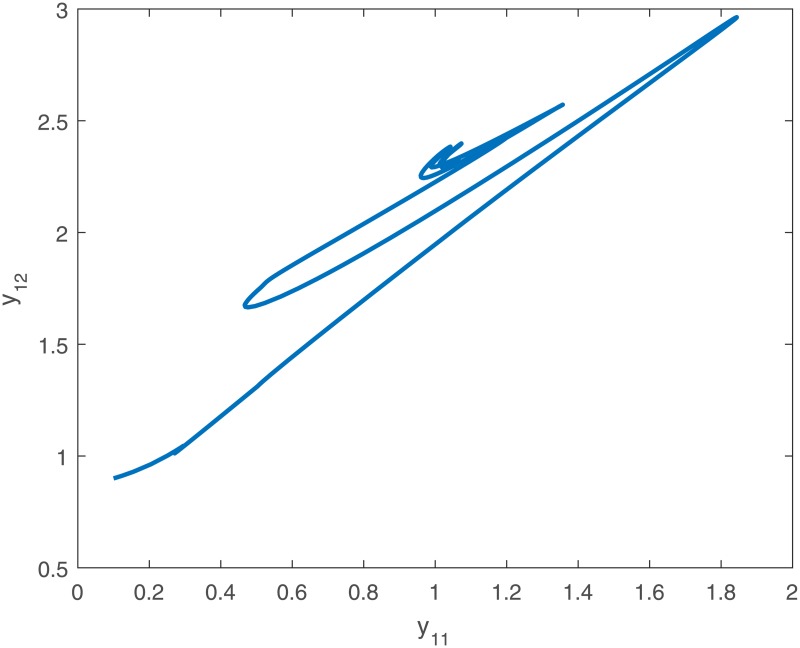
The phase diagram of the first neuron (*y*_11_ − *y*_12_) of the response system (19) with initial conditions *y*_1_(*t*_0_) = [0.1 + *sin*(*t*), *cos*(*t*) − 0.1]^*T*^, *y*_2_(*t*_0_) = [*sin*(*t*) − 0.1, 0.1 + *cos*(*t*)]^*T*^, *y*_3_(*t*_0_) = [0.5 + *sin*(*t*), *cos*(*t*) − 0.5]^*T*^ when no controller (8).

When no controller *u*_*i*_(*t*) is added into the system, the trajectories of the drive system (18) and the response system (19), and the phase diagram of the first neuron for the drive-response systems are shown in Figs 1, 2, 3 and 4.

**Fig 5:**
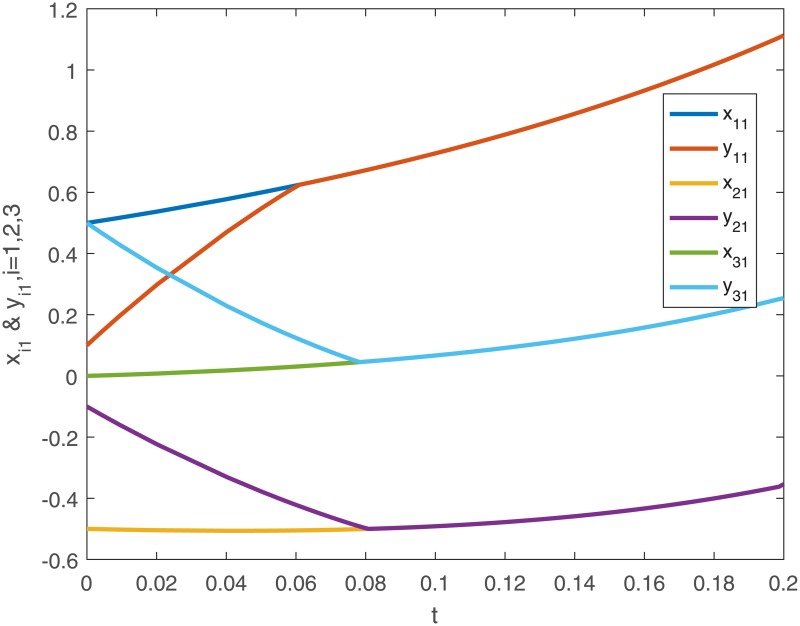
The the first dimensional trajectories of drive-response systems (18) and (19) with initial conditions *x*_1_(*t*_0_) = [0.5 + *sin*(*t*), *cos*(*t*) − 0.5]^*T*^, *x*_2_(*t*_0_) = [*sin*(*t*) − 0.5, 0.5 + *cos*(*t*)]^*T*^, *x*_3_(*t*_0_) = [*sin*(*t*), *cos*(*t*)]^*T*^ and response system (19) with initial conditions *y*_1_(*t*_0_) = [0.1 + *sin*(*t*), *cos*(*t*) − 0.1]^*T*^, *y*_2_(*t*_0_) = [*sin*(*t*) − 0.1, 0.1 + *cos*(*t*)]^*T*^, *y*_3_(*t*_0_) = [0.5 + *sin*(*t*), *cos*(*t*) − 0.5]^*T*^ under the controller (8).

**Fig 6:**
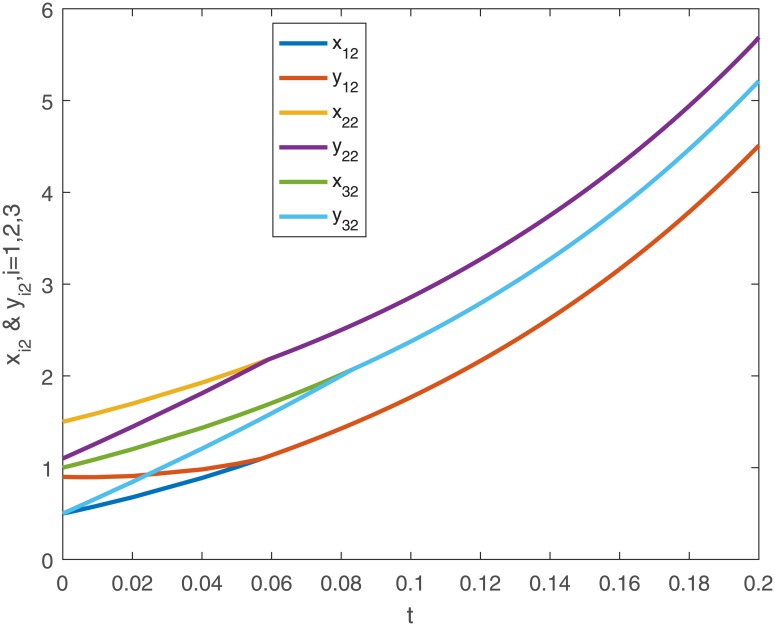
The second dimensional trajectories of with initial conditions *x*_1_(*t*_0_) = [0.5 + *sin*(*t*), *cos*(*t*) − 0.5]^*T*^, *x*_2_(*t*_0_) = [*sin*(*t*) − 0.5, 0.5 + *cos*(*t*)]^*T*^, *x*_3_(*t*_0_) = [*sin*(*t*), *cos*(*t*)]^*T*^ and response system (19) with initial conditions *y*_1_(*t*_0_) = [0.1 + *sin*(*t*), *cos*(*t*) − 0.1]^*T*^, *y*_2_(*t*_0_) = [*sin*(*t*) − 0.1, 0.1 + *cos*(*t*)]^*T*^, *y*_3_(*t*_0_) = [0.5 + *sin*(*t*), *cos*(*t*) − 0.5]^*T*^ under the controller (8).

**Fig 7:**
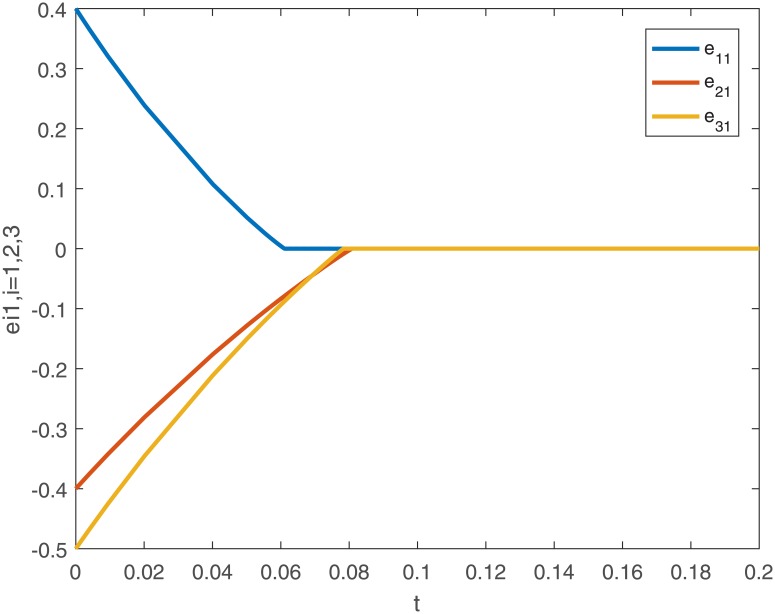
The first dimensional error curves (*e*_*i*1_, *i* = 1, 2, 3) of drive-response systems (18) and (19) with the controller (8).

**Fig 8:**
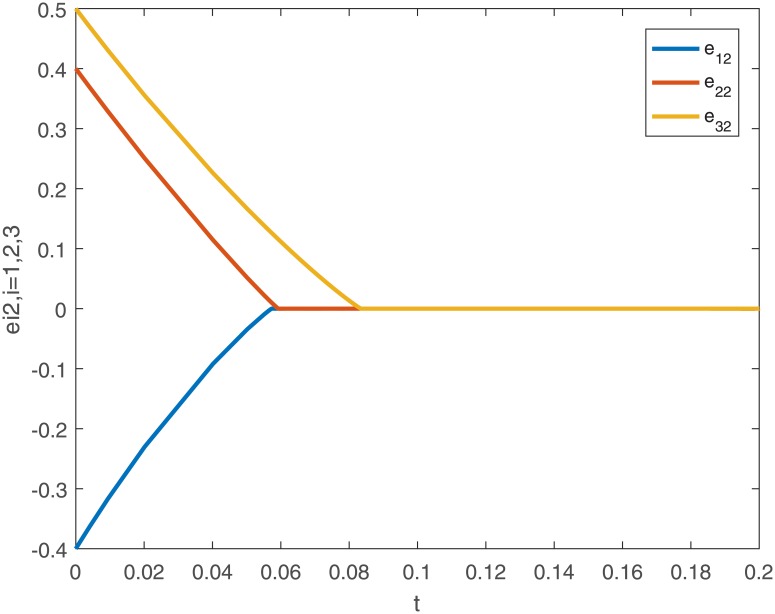
The second dimensional error curves (*e*_*i*2_, *i* = 1, 2, 3) of drive-response systems (18) and (19) with the controller (8).

When using controller *u*_*i*_(*t*), the trajectories and the error curves of the drive-response systems (18) and (19) are shown in the following Figs 5, 6, 7 and 8.

From the Eq (11), we can calculate the settling-time *T*_*max*_ ≈ 1.9740.

*Remark 8*. Obviously, from Figs 1–4, we can see that the drive-response systems (18) and (19) can not reach the synchronization state when the controller (8) is not used. However, when the controller (8) is used, the drive-response systems achieve synchronization, which is easy to see from Figs 5–8. The simulation results verify the effectiveness of **Theorem 1**.

Next, we verify the rightness of *Corollary 4*. through the following example.

*Example 2*: Consider the following two-dimensional drive neutral-type neural network:

$$\begin{array}{c} \begin{array}{cc} \dot{x}\left(t\right) & =D\dot{x}\left(t-\tau_{1}\left(t\right)\right)-Cx\left(t\right)+Af\left(x\left(t\right)\right)+Bg\left(x\left(t-\tau_{2}\left(t\right)\right)\right), \end{array} \end{array}$$ (20)

and the response system as

$$\begin{array}{c} \begin{array}{cc} \dot{y}\left(t\right) & =D\dot{y}\left(t-\tau_{1}\left(t\right)\right)-Cy\left(t\right)+Af\left(y\left(t\right)\right)+Bg\left(y\left(t-\tau_{2}\left(t\right)\right)\right) \end{array} \end{array}$$ (21)

where *τ*_1_(*t*) = 0.2 + 0.5|*sin*(*t*)|, *τ*_2_(*t*) = 0.3 + 0.6|*cos*(*t*)|, *f*(*x*) = *tanh*(*x*), *g*(*x*) = 0.5(|*x* + 1| − |*x* − 1|). Other parameters in the model (20) are chosen as follows:

$$\begin{array}{c} \begin{array}{cc} & D=\text{diag}\left\{0.2,0.2\right\},C=\text{diag}\left\{3.6,3.6\right\},A=[\begin{array}{cc} 0.1 & 0.2\\ 0.3 & 1 \end{array}],B=[\begin{array}{cc} 1.2 & 0.1\\ 0.1 & 1.2 \end{array}], \end{array} \end{array}$$

According to the conditions (16) in *Corollary 4*, the parameters of controller (15) are set as follows:

$$\begin{array}{c} \begin{array}{cc} & \Xi =\text{diag}\left\{1,1\right\},\Omega_{1}=\text{diag}\left\{1,1\right\},\Omega_{2}=\text{diag}\left\{1,1\right\},\\ & K=\text{diag}\{2,2\},R=\text{diag}\{3,3\},\alpha =1.5,\beta =0.5. \end{array} \end{array}$$

The initial conditions of (20) and (21) are *x*(*t*_0_) = [3 + *sin*(*t*), 2 − *cos*(*t*)]^*T*^, *y*(*t*_0_) = [1 − 2*sin*(*t*), 1 + *cos*(*t*)]^*T*^.

**Fig 9:**
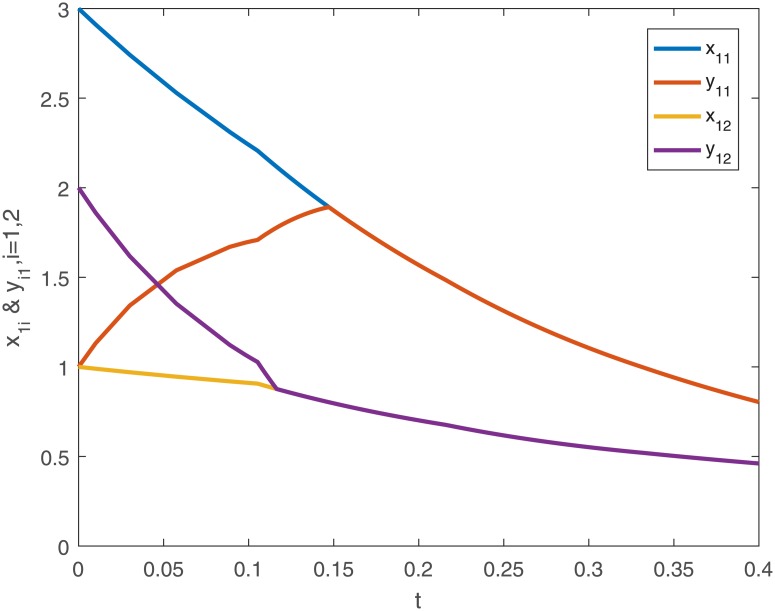
The trajectories of drive-response systems (20) and (21) with initial conditions *x*(*t*_0_) = [3 + *sin*(*t*), 2 − *cos*(*t*)]^*T*^, *y*(*t*_0_) = [1 − 2*sin*(*t*), 1 + *cos*(*t*)]^*T*^ under the controller (15).

**Fig 10:**
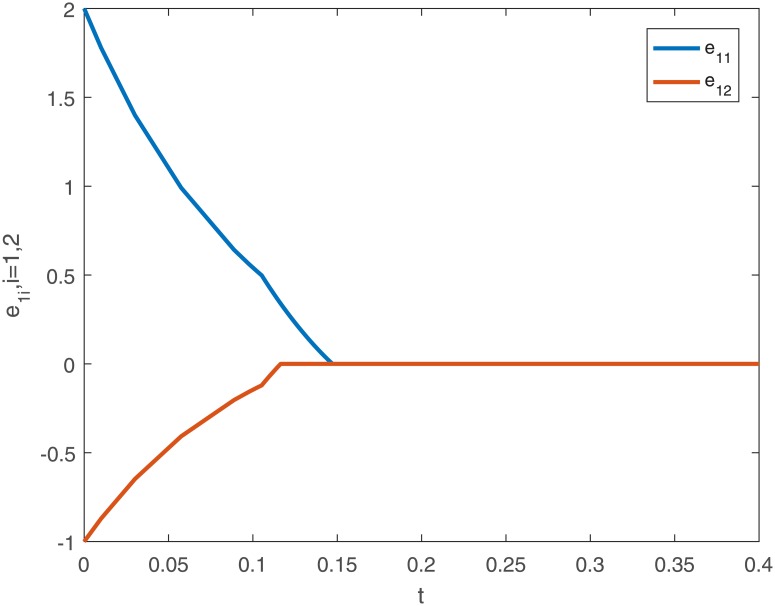
The error curves of drive-response systems (20) and (21) under the controller (15).

The corresponding results are shown in Figs 9 and 10.

Similarly, we can calculate the settling-time *T*_*max*_ ≈ 1.6667 from the Eq (11).

*Remark 9*. The synchronization of nonlinear systems, including neural networks, has many potential practical applications, such as synchronization-based secure communication, signal transmission, automatic control, pattern recognition, etc. In these applications, it is sometimes necessary to accomplish a certain task within a finite/fixed time. For example, in robotics control, we need to drive the robot to reach a specified position at a given time [72]; in a traffic dynamics of signalized intersections network, the intersections must be automatically regulated in a fixed time [73]. Therefore, the study of this paper has some practical significance.

## Conclusion

In this paper, the globally fixed-time synchronization problem of a class of coupled neutral-type neural networks with mixed time-varying delays is studied. The proposed network model is more general than the model of earlier publications. A general discontinuous feedback controller is designed to guarantee the drive-response systems to achieve fixed-time synchronization. By virtue of the definition of fixed-time synchronization, some lemmas, the upper right-hand derivative of discontinuous function, and a simple Lyapunov function, some fixed-time synchronization criteria are obtained through mathematical derivation. Some corollaries about the fixed-time synchronization and some special cases of proposed model have been also given. Finally, the effectiveness of the proposed theorem and corollaries has been validated by two numerical examples. For future research topics, (1) more simple controllers and more easily validated conditions will be studied to guarantee the fixed-time synchronization of neutral-type neural networks or other complex dynamics networks; (2) based on some existing literatures [74], we will consider the problem of fixed-time synchronization of neural-type neural networks with stochastic factors or Markovian jump; (3) considering that the neuron model studied in this paper is to artificial, we will investigate some classical physical-biological models, as shown in Refs. [75, 76].
